# Macrophage Depletion Reduces Disease Pathology in Factor H-Dependent Immune Complex-Mediated Glomerulonephritis

**DOI:** 10.1155/2022/1737419

**Published:** 2022-01-20

**Authors:** Alexander Jacob, Michael Phelps, Shane Fraher, Marcos Lopez, Anthony Chang, Richard J. Quigg, Jessy J. Alexander

**Affiliations:** ^1^Department of Medicine, University at Buffalo, Buffalo, NY 14203, USA; ^2^Department of Pathology, University of Chicago, Chicago, IL 60637, USA

## Abstract

Complement factor H (FH) is a key regulator of the alternative pathway of complement, in man and mouse. Earlier, our studies revealed that the absence of FH causes the C57BL6 mouse to become susceptible to chronic serum sickness (CSS) along with an increase in the renal infiltration of macrophages compared to controls. To understand if the increased recruitment of macrophages (M*ϕ*s) to the kidney was driving inflammation and propagating injury, we examined the effect of M*ϕ* depletion with clodronate in FH knockout mice with CSS. Eight-week-old FHKO mice were treated with apoferritin (4 mg/mouse) for 5 wks and with either vehicle (PBS) or clodronate (50 mg/kg ip, 3 times/wk for the last 3 weeks). The administration of clodronate decreased monocytes and M*ϕ*s in the kidneys by >80%. Kidney function assessed by BUN and albumin remained closer to normal on depletion of M*ϕ*s. Clodronate treatment prevented the alteration in cytokines, TNF*α* and IL-6, and increase in gene expression of connective tissue growth factor (CTGF), TGF*β*-1, matrix metalloproteinase-9 (MMP9), fibronectin, laminin, and collagen in FHKO mice with CSS (*P* < 0.05). Clodronate treatment led to relative protection from immune complex- (IC-) mediated disease pathology during CSS as assessed by the significantly reduced glomerular pathology (GN) and extracellular matrix. Our results suggest that complement activation is one of the mechanism that regulates the macrophage landscape and thereby fibrosis. The exact mechanism remains to be deciphered. In brief, our data shows that M*ϕ*s play a critical role in FH-dependent ICGN and M*ϕ* depletion reduces disease progression.

## 1. Introduction

Chronic kidney disease (CKD) is a global health problem; 10% of which is immune complex- (IC-) mediated and occurs in serum sickness, infections, and autoimmune diseases. [1]. The USA Renal Data System Annual Report states that individuals with CKD “are clearly at a high risk of death^”^ with 10-fold increased incidence over three decades in which affected patients lost 70% of their lifespan. According to the NIDDK, approximately 14% of the adult population has been diagnosed with kidney disease, making it the 9^th^ leading cause of death [2]. The kidney is highly susceptible to complement-mediated damage especially in autoimmune and inflammatory settings [3, 4].

The complement system participates in innate and adaptive immune responses and provides a first line of defense against microorganisms [5, 6]. It consists of three activation pathways, which are initiated in unique ways, and made up of more than 30 plasma and cell-associated proteins [7]. The alternative pathway is constantly on alert and is regulated by proteins such as complement factor H (FH). FH is a circulating protein mostly synthesized by the liver [8]. FH-deficient (fh^−/−^) mice treated with apoferritin develop chronic serum sickness (CSS) leading to diffuse proliferative glomerulonephritis (GN) within 5 weeks of treatment, while littermate C57BL/6 mice that are factor H (FH) sufficient have little glomerular inflammation [9]. F4/80^+^ macrophage infiltration occurs in the kidney and is observed around IC deposits in the fh^−/−^ mice. Complement activation generates anaphylatoxins, C3a and C5a, that participate in trafficking of M*ϕ*s to the site of inflammation. Inhibition of C5a/C5aR1 signaling reduced the M*ϕ*s in the kidney [10]. In this setting, kidney injury is associated with renal infiltration but the mechanisms that may be involved in causing renal injury remains unknown.

Monocytes (Mo) generated by hematopoietic cells [11] migrate through the endothelium into the tissues, where they differentiate into M*ϕ*s. Markers such as F4/80, CD11b, CD68, and Fc receptors can identify the M*ϕ*s. M*ϕ*s are critically involved in renal injury, repair, and fibrosis in different experimental models of renal disease [12, 13]. Recently, our studies demonstrated that the kidney injury in FH-dependent ICGN is associated with the renal infiltration of M*ϕ*s. Moreover, previous studies have demonstrated that inhibition of renal M*ϕ* infiltration reduces arterial pressure and prevents glomerular injury and fibrosis in various animal models of nephropathy [14, 15]. However, to our knowledge, the impact of M*ϕ* depletion during renal injury associated with FH-dependent ICGN has not been addressed. Therefore, the present study examined the role of M*ϕ*s on the renal injury associated with FH-dependent ICGN using liposome clodronate. Clodronate (clodronic acid) is a bisphosphonate that is metabolized to a nonhydrolyzable ATP analog [16]. Our experiments show that M*ϕ*s play a key role in loss of kidney function in a setting of unregulated complement activation. Therefore, reducing M*ϕ*s or instructing M*ϕ*s to assume a repair phenotype could reduce disease pathology and can be exploited therapeutically.

## 2. Materials and Methods

### 2.1. Experimental Animals

The fh^−/−^ mouse strain was generated and kindly provided by Drs. Matthew Pickering and Marina Botto (Imperial College of London) and continuously backcrossed onto the C57BL/6 strain in our laboratory for over 10 generations. Littermate FH-sufficient mice served as the controls (*n* = 8/group) Genotyping was performed using PCR-based approaches. Mice were maintained at a temperature of 20–22°C in 12 h light–12 h dark lighting cycle, in sterile, ventilated cages with food and water *ad lib*. The University of Chicago and University at Buffalo Institutional Animal Care and Use Committees approved all studies.

### 2.2. Chronic Serum Sickness Protocol

CSS was induced in 6–8 week fH^−/−^ or wild-type mice with daily intraperitoneal administration of 4 mg horse spleen apoferritin (Calzyme Laboratories) without adjuvant [10, 17, 18] for 5 weeks. Littermate controls received intraperitoneal injections of saline vehicle contemporaneously.

### 2.3. Kidney Function Assessments

Blood urea nitrogen (BUN) concentrations were determined with a Beckman Autoanalyzer (Beckman Coulter, Fullerton, CA). Urinary albumin concentrations were measured with a mouse albumin ELISA kit (Bethyl Laboratories, Montgomery, TX) and normalized to urinary creatinine measured colorimetrically (Stanbio Laboratory, Boerne, TX) as described previously [19].

### 2.4. Histology

Kidneys were embedded in paraffin and formalin-fixed, and four-micrometer sections were generated. The sections were stained with Periodic Acid-Schiff and examined by a renal pathologist (A.C.) in a blinded manner. For each slide, proliferative glomerulonephritis, glomerulosclerosis was graded from 0 to 4 as described previously [20].

For immunofluorescence microscopy, the kidney tissue was snap-frozen in 2-methylbutane precooled in dry ice. Four-micron cryostat sections were processed for immunofluorescence (IF) staining. Sections were fixed in 4% paraformaldehyde and stained with Alexa488-conjugated anti mouse C3 (Cappel) and Alexa 555 anti-mouse IgG. A semiquantitative score of staining intensity and distribution from 0 to 4 was provided in a blinded manner as described previously [19].

### 2.5. qRT-PCR

The kidneys were harvested, and total RNA was extracted from the tissues using TRIzol reagent according to the manufacturer's instructions (Invitrogen, USA). cDNA was synthesized using SuperScript III Reverse Transcriptase (Invitrogen). *q*RT-PCR was performed using Power SYBR Green PCR Master mix on the ABI 7700 sequence detector (Applied Biosystems). PCR amplification was performed in a total volume of 25 *μ*l containing 12.5 *μ*l of 2x TaqMan Universal PCR Master Mix (Applied Biosystems), 6.25 *μ*l of nuclease-free water, 5 *μ*l of cDNA, and 1.25 *μ*l of the appropriate primers (IDT). All qPCR primers (Table 1) were designed using the Primer3 software (Whitehead Institute for Biomedical Research) to ensure specificity and sensitivity. To quantify the levels of mRNA, we normalized expression of the target genes to glyceraldehyde-3-phosphate dehydrogenase. Relative mRNA expression levels were calculated using the 2 ^− *ΔΔ*Ct^ method and were normalized to the expression levels of GAPDH.

### 2.6. Statistics

All results are mean ± SD from at least 6-8 mice in each group. Either an unpaired two-tailed test (data with normal distribution) or the Mann–Whitney *U* test (data with not normal distribution) was used to compare 2 groups by GraphPad Prism 5.0. The statistical significance is expressed as follows: ^∗^*P* < 0.05; ^∗∗^*P* < 0.01; and n.s.: not significant.

## 3. Results

We induced CSS in mice by repetitive immunization with horse spleen apoferritin. Animals generate an active antiapoferritin IgG humoral immune response and deposit IgG in glomeruli. Our earlier studies revealed that there is increased M*ϕ* infiltration into the kidneys in this setting.

### 3.1. Macrophage Gene Expression Is Increased in FH-Dependent ICGN Kidneys

**Figure 1:**
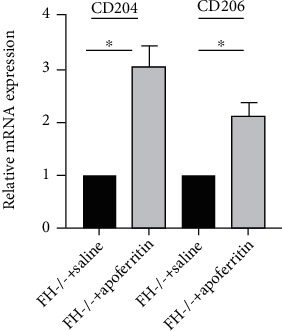
FH alters macrophage marker genes in the kidney of mice with FH-dependent ICGN. Real-time quantitative PCR for *CD204* and *CD206* using mRNA isolated from the kidneys after 5 weeks of saline or apoferritin. The expression is normalized to *GAPDH* (*n* = 6/group). ^∗^*P* < 0.01. Graphs depict fold change of genes in apoferritin-treated fh^−/−^ mice (*n* = 6) compared to fh^−/−^ mice treated with saline (*n* = 6).

To evaluate the presence of M*ϕ*s in FH-dependent ICGN kidneys, qRT-PCR was performed with M2 macrophage markers, CD204 (M*ϕ* scavenger receptor 1) and CD206 (M*ϕ* mannose receptor 1) [21–24]. Expression of both genes in kidney was significantly upregulated following apoferritin treatment in FHKO mice (Figure 1) indicating increased presence of macrophages in the kidney.

### 3.2. Time-Course Changes by Chlodronate Treatment in Renal Macrophage Infiltration in Apoferritin-Treated FHKO Mice

**Figure 2:**
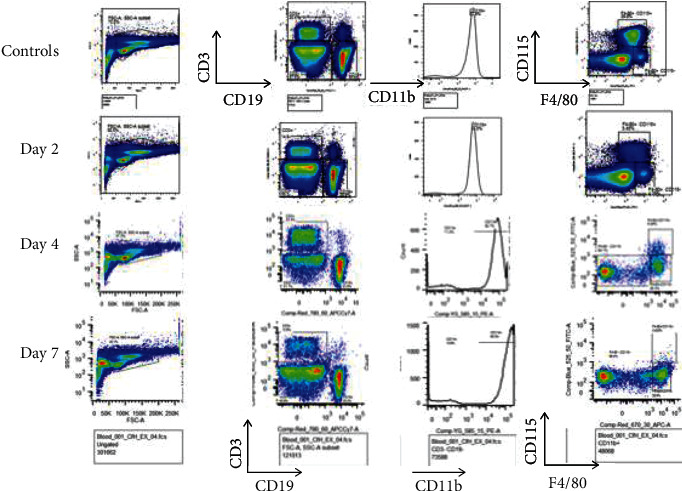
Clodronate increasingly reduces kidney macrophages in mice with FH-dependent ICGN. Given are the flow cytometry profiles of F4/80+ monocytes/M*ϕ*s on day 0, 2, 4, and 7, representatively. The expression of F4/80+ cells was determined by flow cytometry. Data were analyzed using FlowJo V10 software. F4/80+ cells are significantly reduced at each time point reaching less than 10% by day 7. Each time point *n* = 6.

**Figure 3:**
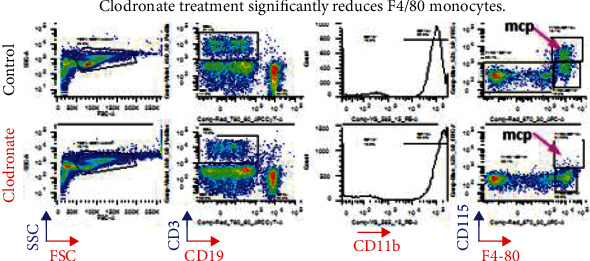
Supplemental data. Given are the flow cytometry profiles of F4/80+ monocytes from the control and clodronate-treated mice. F4/80+ cells are significantly reduced reaching less than 10% by clodronate treatment. Each time point *n* = 6.

To determine the effective method of reducing M*ϕ*s in FH-dependent ICGN, we depleted M*ϕ*s in fh^−/−^ mice treated with CSS by clodronate liposome treatment. Mice were treated with clodronate liposome or control liposome every 3 days for 3 weeks. The time-course changes in renal M*ϕ* infiltration in apoferritin-treated FHKO mice are shown in Figures 2 and 3. Renal M*ϕ* infiltration in the kidneys was reduced when assessed every alternate day and was a negligible presence (less than 10% similar to that observed by Kim et al. [25]) by day 7 in apoferritin-treated FHKO mice.

### 3.3. Macrophage Depletion Prevents Aberrant Kidney Function in FH-Mediated ICGN

**Figure 4:**
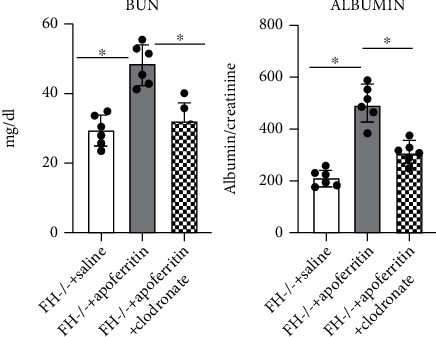
M*ϕ* depletion prevents reduced kidney function in FH-dependent ICGN. Shown are final blood urea nitrogen (BUN) and albumin concentrations from fH^−/−^ mice treated with saline (control), apoferritin (ICGN), or apoferritin+clodronate. Each circle is from an individual animal. ^∗^*P* = 0.015 versus the control.

The kidney function in the different mice groups was analyzed by determining serum BUN and urine albumin. Urine and blood were collected at the time of sacrifice. Serum BUN and urine albumin were increased in FHKO mice but remained closer to normal in those mice treated with clodronate Figure 4. Depletion of M*ϕ*s did not change the antiapoferritin in circulation (results not shown).

### 3.4. Macrophage Depletion Reduces Glomerular Pathology in FH-Mediated ICGN

**Figure 5:**
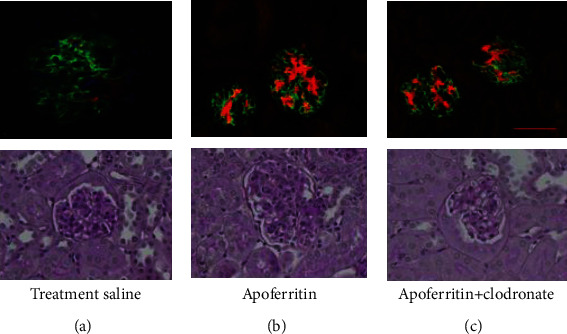
M*ϕ*s depletion reduces glomerular pathology in FH-dependent ICGN. Given are representative images of glomeruli from saline-treated (a), apoferritin-treated (b), and apoferritin+clodronate-treated FH-deficient mice. In (A), the sections are stained with C3 (green) and IgG (red), while (B) show sections stained with PAS. IgG deposits that were increased with apoferritin treatment remained unchanged, while the glomeruli looked closer to normal on macrophage depletion.

Immune complex deposits in the kidney and histopathological features of ICGN were evaluated at the end of the experiment. Immunofluorescence staining for C3 and IgG revealed that M*ϕ* depletion did not modulate the immune complexes in this setting. Representative immunofluorescence staining and histopathology are shown in Figure 5. The primary histopathological feature was of diffuse hypercellularity of the glomerular tufts in fh^−/−^ mice with CSS was significantly reduced on depletion of M*ϕ*s.

### 3.5. Macrophages Modulate Cytokines in FH-Dependent ICGN

**Figure 6:**
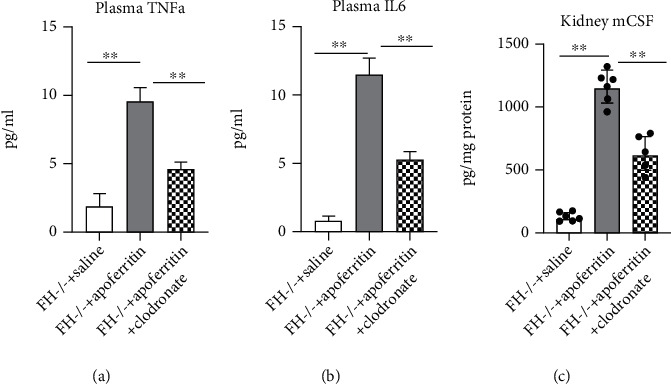
M*ϕ*s modulate cytokines in FH-dependent ICGN. The plasma and kidneys obtained at the time of sacrificed were stored at -80°C and processed as given in Materials and Methods. The concentrations of plasma TNF*α* (a), plasma IL-6 (b), and kidney M-CSF (c) were determined by ELISA. Results are representative of multiple experiments. ^∗^*P* < 0.05, ^∗∗^*P* < 0.01, and ^∗∗∗^*P* < 0.001.

Once M*ϕ*s infiltrate the kidney and are exposed to inflammatory stimuli, they liberate cytokines. We evaluated, by ELISA, plasma circulating levels of TNF*α* and IL-6 and the kidney mCSF in FHKO mice treated with saline, apoferritin, or apoferritin and clodronate. The circulating levels of IL-6 were very low in wild-type mice and increased in FHKO mice and were reduced in FHKO mice treated with clodronate (Figure 6). The three cytokines, TNF*α*, IL-6, and mCSF were significantly increased in mice treated with apoferritin compared to saline-treated controls. M*ϕ* depletion by clodronate significantly limited the rise in cytokines in this setting.

### 3.6. Macrophages Modulate Profibrotic Molecules and Laminin in FH-Dependent ICGN

**Figure 7:**
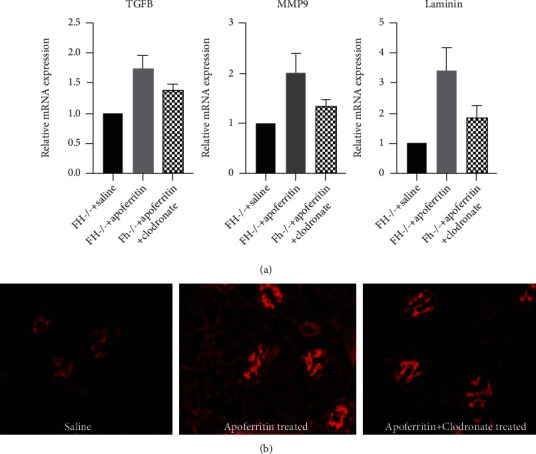
Laminin deposition in FH-deficient kidneys with ICGN is reduced by M*ϕ*s depletion. (a) qRT-PCR for TGFB, MMP9, and laminin from the kidney mRNA isolated from mice after 5 weeks of saline and apoferritin and 3 weeks of clodronate. The expression is normalized to *GAPDH* (*n* = 6/group). (b) Representative IF photomicrographs for laminin staining in the kidneys of the same groups of mice. ^∗^*P* < 0.01.

Once the kidneys develop a disease, they tend to become fibrotic. M*ϕ*s participate in the fibrotic process by overproducing profibrotic molecules. Real-time PCR revealed a 75% increase in transforming growth factor-beta1 (TGFbeta1) mRNA expression, a 100% increase in metalloproteinase-9 (MMP9) expression and 230% increase in laminin mRNA. In line with the mRNA expression, laminin protein expression is significantly increased in the fh^−/−^ mice with CSS which was reduced in the absence of M*ϕ*s (Figure 7).

## 4. Discussion

Our recent studies have shown that in the absence of FH, mice develop diffuse proliferative glomerulonephritis (GN) with F4/80^+^ M*ϕ*s around sites of ICs [10]. Furthermore, our studies demonstrate that Cd11b^−/−^fh^−/−^ mice have exacerbated features of glomerulonephritis [26]. Given that the presence of increased inflammatory M1 macrophages and their sustained activation could cause tissue pathology, and their repolarization into the anti-inflammatory phenotype would help in resolution of disease pathology, the current study was undertaken to further understand the role of M*ϕ*s in fh^−/−^ mice with ICGN. The increased presence of M*ϕ*s in the kidney and the resolution of disease pathology by depletion of macrophages during FH-mediated ICGN demonstrate the critical role of M*ϕ*s in this setting, and therefore, they could serve as important therapeutic targets in this setting.

Absence of FH leads to increased C5a production. C5a binds to C5aR1 signaling Mos to the kidney where they add to the inflammatory milieu. In addition, our studies also demonstrated that in this setting of excessive C5a generation, inactivation of C5a appears to limit the disease [10]. Activation of Mos causes them to become polarized, but they remain in a state of continuum [27]. Our results in this study show that glomerular disease in CSS was reduced in fh^−/−^ mice in which M*ϕ*s were depleted compared to fh^−/−^ mice without macrophage depletion. Thus, eliminating M*ϕ*s alleviated ICGN in fh^−/−^ mice reducing the disease pathology. However, M*ϕ*s are also necessary for the repair process, and since M*ϕ*s change states from pro- to anti-inflammatory states and vice versa, it will be our future endeavor to understand whether altering the M*ϕ*s landscape without depleting them could alter the disease pathology.

M*ϕ*s play an important role in the initiation and maintenance of fibrosis and are also involved in the suppression, resolution, and reversal of fibrosis [28]. Our results show increased expression of CD204+ and CD206+ M*ϕ* markers suggesting their increased presence in this setting. Since these M*ϕ*s are a major source of profibrotic molecules, our results showing increase in TGF*β*-1 as well as MMP9 suggest that these M*ϕ*s could be contributing both directly and indirectly to fibrotic processes in FH-mediated ICGN through different mechanisms (1,3,8,44). TGF*β*-1 can alter fibrosis by cytokine regulation. In addition, TGF*β*-1 modulates C3 and factor B biosynthesis and expression in monocytes [29], and alteration of complement in the immune cells and kidney cells can facilitate tissue fibrosis [30]. However, the exact interactions between macrophages and complement, the mechanism/s involved, their roles, and tissue compartmentalization need to be deciphered [31]. Our studies showed reduced expression of laminin in M*ϕ*s-depleted mice compared to M*ϕ*-sufficient mice. To better understand how FH regulates the fibrotic process, we need to identify the specific M*ϕ* subsets that are present in FH-mediated ICGN and elucidate the contributions of each subset population. These studies are important since complement therapeutics has become a clinical reality. Inhibitors have been and continue to be developed against different targets in the complement cascade that will block the downstream steps in the in the activation pathway(s). Currently, one of the complement inhibitor in clinical use is the anti-C5 antibody, Eculizumab [32]. Therefore, identifying the pathogenic complement components in the disease and supplementing that with regulation of the M*ϕ*s population is not a trivial point.

In summary, here we studied the role of M*ϕ*s in the FH-dependent ICGN model. The results can also be applied in settings of unregulated complement activation with immune complexes such as those observed in different diseases including lupus nephritis, IgA nephropathy, and membranous nephropathy. We show the importance of both complement regulation by FH and the M*ϕ*s status in the disease. Thus, pharmacological targeting of the complement cascade and the M*ϕ*s status in IC-mediated GN has potential clinical relevance.

## Figures and Tables

**Table 1:** Primer sequences used to assess gene expression by qRT-PCR.

Gene	Forward primer sequence	Reverse primer sequence
GAPDH,	5′-TTGATGGCAACAATCTCCAC-3′	5′- CGTCCCGTAGACAAAATGGT-3′
*Markers*:		
CD206	5′-CAGGAGGAGAGAGATCCGATTTA-3′	5′- GCATTAGCATGGAAGCAAAGA-3′
CD204	5′ TGGAGGAGAGAATCGAAAGCA-3′	5′- CTGGACTGACGAAATCAAGGAA-3′
Matrix proteins:		
TGF-*β*1	5′- TGCGCTTGCAGAGATTAAAA-3′	5'-AGGTAACGCCAGGAATTGTTGCTA-3′
MMP9	5′-CTTCTGGCGTGTGAGTTTCCA-3′	5′-ACTGCACGGTTGAAGCAAAGA-3′
Laminin,	5′-CGGAGCCCTGCATCACAAA -3′	5′- AGCAAGGTCGTCCTCAAAGC -3′

ELISA: serum TNF*α* and IL-6 levels were measured by ELISA MAX™ Standard Kits (BioLegend, CA, USA) according to the manufacturer's protocols. The concentrations of IL-1B, M-CSF, and GM-CSF were measured by ELISA (R&D Systems, UK) according to the manufacturer's instructions.

## Data Availability

All data generated during this study are included in this published article.
